# In vitro and in vivo anti-parasitic activity of curcumin nanoemulsion on *Leishmania major* (MRHO/IR/75/ER)

**DOI:** 10.1186/s12906-024-04522-1

**Published:** 2024-06-18

**Authors:** Keivan Sahebi, Fatemeh Shahsavani, Fatemeh Mehravar, Gholamreza Hatam, Rasoul Alimi, Amirhossein Radfar, Mohammad Saleh Bahreini, Ali Pouryousef, Aref Teimouri

**Affiliations:** 1 Student Research Committee, Shiraz University of Medical Sciences, Shiraz, Iran; 2 Department of Parasitology and Mycology, School of Medicine, Shiraz University of Medical Sciences, Shiraz, Iran; 3 Department of Epidemiology and Biostatistics, School of Health, Torbat Heydariyeh University of Medical Sciences, Torbat Heydariyeh, Iran

**Keywords:** Curcumin, Nanoemulsion, Nanoparticles, Anti-leishmanial activity, *Leishmania major*

## Abstract

The present study aimed to assess the anti-leishmanial effects of curcumin nanoemulsion (CUR-NE) against *Leishmania major* (MRHO/IR/75/ER) in both in vitro and in vivo experiments. CUR-NE was successfully prepared *via* the spontaneous emulsification method. The in vitro effect of various concentrations of CUR-NE against *L. major* promastigotes was assessed using the flow cytometry method. In vivo experiments were carried out in BALB/c mice inoculated subcutaneously with 2 × 10^6 ^*L. major* promastigotes. Mice were treated with topical CUR-NE (2.5 mg/ml), intra-lesion injection of CUR-NE (2.5 mg/ml), topical CUR suspension (CUR-S, 2.5 mg/ml), topical NE without CUR (NE-no CUR), amphotericin B as the positive control group, and infected untreated mice as the negative control group. In vitro exposure of promastigotes to CUR-NE showed a dose-dependent anti-leishmanial effect, with a 67.52 ± 0.35% mortality rate at a concentration of 1250 µg/ml and an IC50 of 643.56 µg/ml. In vivo experiments showed that topical CUR-NE and CUR-S significantly decreased the mean lesion size in mice after four weeks from 4.73 ± 1.28 to 2.78 ± 1.28 mm and 4.45 ± 0.88 to 3.23 ± 0.59 mm, respectively (*p* = 0.001). Furthermore, CUR-NE significantly decreased the parasite load in treated mice compared with the negative control group (*p* = 0.001). Results from the current study demonstrated the promising activity of CUR-NE against *L. major* in both in vitro and in vivo experiments. Moreover, CUR-NE was more efficient than CUR-S in healing and reducing parasite burden in mouse models. Future studies should aim to identify molecular mechanisms as well as the pharmacologic and pharmacokinetic aspects of CUR-NE.

**Supplementary Information:**

The online version contains supplementary material available at 10.1186/s12906-024-04522-1.

## Introduction

Leishmaniasis is a zoonotic disease caused by intracellular parasites of the genus *Leishmania*. Annually, more than two million new cases of human infections by *Leishmania* spp. are reported worldwide [1]. The disease has a broad spectrum of clinical manifestations, ranging from a simple to diffused skin ulcer (cutaneous leishmaniasis [CL]), mucosal involvement (mucocutaneous leishmaniasis [MCL]), or spreading to the reticuloendothelial system (visceral leishmaniasis [VL]) [2]. CL is the most common and benign form of leishmaniasis, mostly reported from Afghanistan, Algeria, Iraq, Pakistan, and Syria (the Old World), and Brazil, Colombia, and Peru (the New World) [3]. The disease is primarily caused by *L. major*, *L. tropica,* and *L. aethiopica* in the Old World [4], while in the New World, *L. mexicana* and parasites from the *Viannia* complex, such as *L.V. braziliensis,* are the primary etiologies [5]. Currently, the main treatment options for leishmaniasis include local intra-lesion injection of glucantime with or without cryotherapy or systemic administration of mitelfosine, pentavalent antimonials (e.g., pentostam and glucantime), pentamidine, or amphotericin B [6]. However, the utility of these treatments is limited by their various side effects, toxicities, and inability to eradicate the parasite [7]. In addition, recent studies have reported drug resistance, even to antimonial compounds, among *Leishmania* spp. [8].

Recently, researchers have considered natural medicines as potential novel antimicrobial agents [9–11]. Curcumin (CUR) is a hydrophobic polyphenol and the active component of the turmeric plant, *Curcuma longa* [12]. CUR has a wide range of biological and pharmacological properties, including antioxidant, anti-inflammatory, and antimicrobial activities [12]. Studies have proven the anti-parasitic activity of CUR against various helminths and protozoa, such as *Schistosoma japonicum*, *S. mansoni* [13], *Leishmania* spp. [14–16], *Toxoplasma gondii* [17], *Trypanosoma* [18, 19], *Plasmodium falciparum* [20, 21], *Giardia lamblia* [22], and *Cryptosporidium parvum* [23]. Nonetheless, due to the hydrophobic nature of CUR, the compound has a very low water solubility [12]. By incorporating CUR into nano-based formulations, nanomedicine has greatly aided in improving CUR’s solubility, bioavailability, and stability, as well as the sustained release of the drug to the target tissue [24]. Various formulations have been described to enhance the bioavailability of CUR. Of these formulations, nanoemulsions (NE) have extensively been developed to improve the solubility of CUR [25]. Different CUR nano-formulations have shown more effective anti-leishmanial activity against *L. major* [26, 27], *L. tropica* [28], and *L. donovani* [29, 30]. In our previous works, we have successfully prepared and characterized CUR nanoemulsion (CUR-NE) and addressed its potential anti-parasitic activity against *T. gondii* [17] and *Echinococcus granulosus* [31]. To the best of the author’s knowledge, there have been no prior investigations exploring the effects of CUR-NE against *L. major*. Consequently, this study was designed to evaluate the potential anti-leishmanial activity of CUR-NE on *L. major* through in vitro and in vivo experiments.

## Material and method

### Compounds and parasite strain

CUR (CAS-No:458-37-7; Sigma-Aldrich; Purity: ≥80%), heat-inactivated fetal bovine serum (FBS), RPMI 1640 medium, propidium iodide (PI), penicillin-streptomycin (pen/strep), and soybean oil were purchased from Sigma-Aldrich, USA. Polysorbates of Tween 80 and Tween 85, sodium chloride, ethanol, methanol, and phosphate-buffered saline (PBS) solution were purchased from Merck, Germany. The *L. major* promastigotes (MRHO/IR/75/ER) were provided by the Department of Parasitology, Shiraz University of Medical Sciences, Shiraz, Iran.

### Animals

Thirty female BALB/c mice, weighing 23 ± 2 g and 6–8 weeks old, were purchased from the Comparative and Experimental Medicine Department of Shiraz University of Medical Sciences, Shiraz, Iran. Mice were housed at 25 °C with 12-hour light/dark cycles. Animals had free access to water and food. The study was approved by the Ethics Committee of Shiraz University of Medical Sciences, Shiraz, Iran (ethical code: IR.SUMS.MED.REC.1400.034). Animal care and experiments were carried out according to Guidelines for the Care and Use of Laboratory Animals published by the United States National Institutes of Health and approved by the Ethical Committee of Shiraz University of Medical Sciences, Shiraz, Iran.

### Preparation and characterization of curcumin nanoemulsion

As illustrated in our previous work [31], the CUR-NE was prepared using the spontaneous emulsification method. In brief, CUR was dissolved in the oil phase (soybean oil) by slowly adding surfactants (Tween 80 and 85), co-surfactant (ethanol), and distilled water (DW). Particle size, zeta potential, and morphology were characterized using the Malvern Zetasizer Nano ZS instrument (Malvern, UK) and transmission electron microscopy (TEM). The stability of CUR-NE was assessed by the determination of particle size and zeta potential after three freeze-thaw cycles. The cytotoxicity of CUR-S, CUR-NE, and NE-no CUR was evaluated in our previous study [32], with 50% inhibitory concentrations (IC50) of 1495.66 µg/ml, 3222.7 µg/ml, and 1832.53 µg/ml, respectively.

### Parasite culture

RPMI 1640 medium, supplemented with 10% heat-inactivated FBS and 1% pen/strep, was used for mass culture of *L. major* promastigotes. Cultures were incubated at 24 ± 1 °C, and the promastigotes were transferred weekly from previous media into fresh cultures.

### In vitro experiments

The flow cytometry method using the BD FACSCalibur Flow Cytometer (BD Biosciences, USA) was employed for the assessment of the anti-parasitic activity of CUR-NE against *L. major* promastigotes. According to previously established methods [33], the promastigotes in the late logarithmic phase were used for the assay. The gates were determined based on the scatter properties of the cells (the side scatter [SSC] and forward scatter [FSC] signals), and the cell count was finalized at 10,000 cells. The assay was performed using a 488 nm-wavelength laser.

First, 2 × 10^5^ promastigotes were added to eight tubes. Five tubes contained 78, 156, 312, 625, and 1250 µg/ml of CUR-NE, and one tube contained NE without curcumin (NE-no CUR). The positive control (PC) tube contained 20 µg/ml amphotericin B, and the negative control (NC) tube contained only promastigotes. After three hours of incubation at 24 ± 1 °C, all tubes were stained with 50 µg/ml of PI dye for 15 min. According to the methods described for *L. major* promastigotes [33], the flow cytometry was performed in duplicates. Using statistical analysis, the IC50 value was calculated.

### In vivo experiments

Using a subcutaneous injection of 2 × 10^6^ infective *L. major* promastigotes (MRHO/IR/75/ER) into the base of the mice’s tails, thirty mice were successfully infected. Four weeks after the initial infection, cutaneous lesions were established at the base of the mice’s tails, and mice with similar lesion sizes were selected and entered the study. Chosen mice were then divided into six groups (*n* = 5 per group) as follows: group 1 consisted of mice treated with intra-lesion injection of 0.5 ml of CUR-NE (2.5 mg/ml); group 2 consisted of mice treated topically with 0.5 ml of CUR-NE (2.5 mg/ml); group 3 consisted of mice treated topically with 0.5 ml of CUR suspension (CUR-S, 2.5 mg/ml); group 4 consisted of mice treated topically with NE-no CUR; group 5 consisted of mice treated with intra-lesion injection of 2 mg/kg amphotericin B, as the PC group; and group 6 consisted of infected untreated mice as the NC group. Treatment doses were determined based on the previous studies [26, 34], CUR load in the NE formulation, and drug efficiency in the pilot study. The topical treatments were applied daily to the lesions (without any dressing), followed by keeping the animals 10–15 min immobile.

### Treatment evaluation

All groups, except for the NC group, received their assigned treatment for three weeks. Mice were monitored daily for 28 days, and lesion sizes were documented for each group weekly. The effects of the different treatments on cutaneous lesions were evaluated based on the changes in the lesion sizes using a caliper tool (Mitutoyo, Taiwan). The mean lesion size was calculated as the average of the vertical and horizontal diameters. Measurements were performed on five occasions: day 0 (before initiation of treatments), 7, 14, 21, and one week after the completion of the treatment (day 28).

### Parasite load

To determine the parasite load, smears from the margins of lesions were prepared before and after four weeks of treatment. Slides were fixed with absolute methanol, stained with Giemsa, and evaluated for amastigote load using a Zeiss light microscope (Carl Zeiss, Germany). According to the World Health Organization (WHO) manual [35], parasite loads were determined as 4+ (1–10 parasites/1 field), 3+ (1–10 parasites/10 fields), 2+ (1–10 parasites/100 fields), and 1+ (1–10 parasites/1000 fields).

### Statistical analysis

SPSS software v.21 (IBM Analytics, USA) was used to analyze the data. Mean, standard deviation (SD), median, and interquartile range (IQR) were used to describe the data. Differences in the mean lesion sizes between and within the groups were calculated using the Kruskal-Wallis test and the Mann-Whitney test. The IC50 was calculated using linear regression analysis. *P* values less than 0.05 were considered statistically significant.

## Results

### In vitro experiments

The flow cytometry revealed significant differences in the mortality rates of *L. major* promastigotes exposed to different treatments (Table 1). The NC tube showed an 11.76 ± 0.51% mortality rate, indicating a relatively low death rate of the promastigotes in the absence of any leishmanicidal agent. In contrast, the results of the PC tube demonstrated a substantial mortality rate of 76.91 ± 2.47%, validating the effectiveness of the assay and the susceptibility of *L. major* promastigotes to amphotericin B. In a dose-dependent manner, *L. major* promastigotes treated with different concentrations of CUR-NE (78, 156, 312, 625, and 1250 µg/ml) showed 31.5 ± 5.28, 35 ± 3.75, 44.56 ± 3.02, 48.15 ± 2.48, and 67.52 ± 0.35% mortality rates, respectively (Table 1). Based on statistical analysis, the IC50 for CUR-NE was calculated at 643.56 µg/ml. In addition, exposure of *L. major* promastigotes to NE-no CUR revealed a negligible (17.35 ± 1.03%) anti-leishmanial effect for unloaded NE (Table 1).

**Table 1:** The mean mortality rates of *L. major* promastigotes in exposure to different treatments, using the flow cytometry method

Groups		Mortality rate (%) [Mean (SD)]^*^
Curcumin nano-emulsion concentrations (µg/ml)	78	31.5 (5.28)
	156	35 (3.75)
	312	44.56 (3.02)
	625	48.15 (2.48)
	1250	67.52 (0.35)
Nano-emulsion without curcumin		17.35 (1.03)
Negative control		11.76 (0.51)
Positive control		76.91 (2.47)

*Assays are performed in duplicates

### In vivo experiments

#### Lesion size

**Fig. 1:**
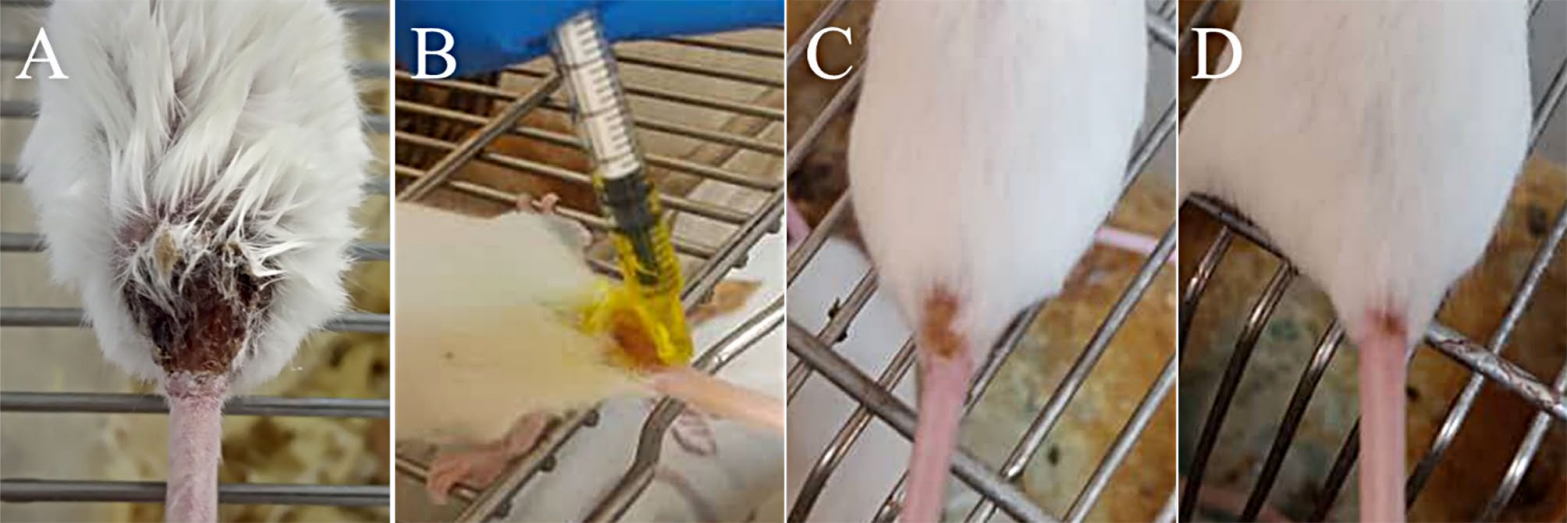
Cutaneous lesions of BALB/c mice infected with *L. major*. (**A**) Control group on the last days of the experiment; (**B**) Topical administration of CUR-NE (week 2); (**C**) CUR-NE (topical) group (week 3); (**D**) CUR-NE (topical) group on the last days of the treatment. CUR-NE: curcumin nano-emulsion

**Fig. 2:**
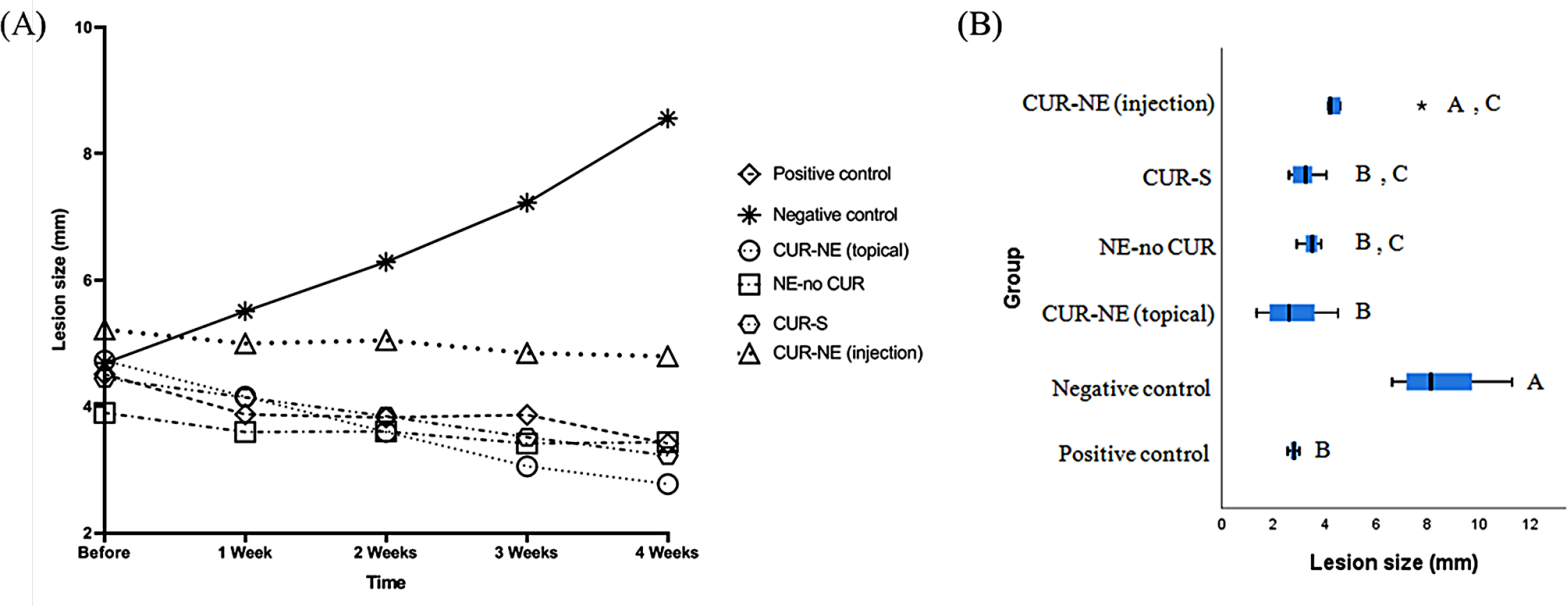
(**A**) Chronological course of mean lesion size over the four-week study period. (**B**) Box plot depicting lesion sizes at week four. Statistically significant differences between groups are denoted by different letters. CUR-NE: curcumin nano-emulsion; NE-no CUR: nano-emulsion without curcumin; CUR-S: curcumin suspension

Four weeks post-inoculation, cutaneous lesions were established at the base of the mice’s tails. Prior to treatment initiation, all selected mice had comparable cutaneous lesion sizes (*p* = 0.314). The mean, SD, median, and IQR of lesion sizes in the six experimental groups are provided in the supplementary material (Table S1). The mean lesion size in the NC group increased progressively after four weeks and reached 8.56 ± 1.90 mm (*p* < 0.001), whereas in the PC group, it demonstrated a remarkable reduction from 4.37 ± 0.06 (before treatment) to 2.78 ± 0.23 mm (week 4) (*p* = 0.022). We showed that cutaneous lesions treated with NE-no CUR did not significantly decrease after four weeks of treatment (*p* = 0.18). Figure 1 shows the therapeutic effect of CUR-NE on lesion size in different weeks of the experiment, compared with that in untreated mice. Topical administration of CUR-NE was capable of decreasing the mean lesion size from 4.73 ± 1.28 (before treatment) to 2.78 ± 1.28 mm (week 4) (*p* = 0.001), which was comparable with the results of the PC group (Fig. 2B). CUR-S as a topical treatment showed some healing effects and decreased the mean lesion size from 4.45 ± 0.88 (before treatment) to 3.23 ± 0.59 mm (week 4) (*p* = 0.001). Intra-lesion injection of CUR-NE, however, was unable to cause a statistically significant decrease in the mean lesion size after four weeks of treatment (*p* = 0.066). A comparison of the lesion sizes in the experimental groups is provided in Fig. 2.

#### Parasite load

Smears before and after four weeks of treatment were studied for the determination of parasite load (Table 2). Amastigote numbers in the NC and NE-no CUR groups did not change after four weeks. Mice in the CUR-NE (topical), CUR-S, CUR-NE (injection), and PC groups exhibited a significant reduction in amastigote load after four weeks of treatment (*p* = 0.001).

**Table 2:** Parasite load reduction in BALB/c mice before and after treatment compared with the negative control group

Group	Parasite load		
	Before treatment	After four weeks	*P* value
CUR-NE (topical)	4+	2+	0.001*
CUR-NE (injection)	4+	3+	0.001*
CUR-S	4+	3+	0.001*
NE-no CUR	4+	4+	1
Negative control	4+	4+	1
Positive control	4+	2+	0.001*

Abbreviations: CUR-NE: Curcumin nanoemulsion, NE- no CUR: Nanoemulsion without curcumin, *significant at *P* < 0.05 compared with the negative control group

## Discussion

CUR is a potent but poorly water-soluble leishmanicidal agent. The compound has been shown to be active against promastigotes of various *Leishmania* spp., including *L. major, L. tropica,* and *L. infantum* [14]. However, due to different extraction methods, the effectiveness and lethal concentrations may vary among studies. For example, Saleheen et al. reported the IC50 values of 4.5, 5.7, and 5.9 µM for *L. major*, *L. tropica*, and *L. infantum*, respectively [14]. Another study by Koide et al. concluded that CUR inhibits 50% of the growth of *L. major* promastigotes at a concentration of 37.6 µM [36]. CUR was able to inhibit *L. amazonensis* promastigotes at a LD50 of 9 µg/ml, while methyl-CUR was the most active derivative with a LD50 of less than 5 µg/ml [16]. Additionally, CUR, demetoxy-CUR, and bisdemetoxy-CUR isolated from *C. longa* demonstrated moderate anti-parasitic activity against *L. major* promastigotes, with IC50s of 7.8, 14.1, and 21.5 µg/ml, respectively [37].

Nano-delivery systems have been of interest to researchers to improve the solubility, bioavailability, and tissue uptake of drugs [24]. Various CUR nano-formulations have been developed and demonstrated better anti-leishmanial activities. Building upon the well-established anti-leishmanial properties of silver, Badirzadeh et al. [26] synthesized CUR-silver nanoparticles (CUR-AgNPs). This approach leverages the potential for reducing silver dosage and improving treatment outcomes. The CUR-AgNPs had promising leishmanicidal activities against *L. major* promastigotes and amastigotes, with an IC50 of 58.99 µg/ml and an EC50 of 58.99 µg/ml. A co-loaded nano-formulation of CUR and miltefosine, using a single-emulsion/solvent evaporation method, exhibited synergistic anti-leishmanial activity in both in vitro and in vivo against *L. donovani* promastigotes and amastigotes [29]. The combination therapy of nanotized CUR and miltefosine facilitated phagocytic activity, reactive oxygen species (ROS) production, and lymphocyte proliferation. Additionally, Fattahi et al. showed that nanoliposomes loaded with CUR were active against *L. major* promastigotes, with an IC50 of 2.33 µg/ml at 72 h [27]. Mannose-conjugated CUR-chitosan nanoparticles (CUR-MCN) were able to inhibit the splenic burden of *L. donovani* in hamsters (94.2%), compared to CUR (2.07%) [30].

From these formulations, NEs have been established as effective and practical technologies to enhance the solubility and targeted delivery of CUR [26–30]. NEs effectively protect the incorporated drug from degradation and often have better stability compared to conventional emulsions [38]. The high negative surface charge of NE particles prevents droplet aggregations and solution instability [31]. Azami et al. investigated the therapeutic effects of CR-NE on acute and chronic toxoplasmosis [17]. Due to systemic delivery, the study employed a higher dosage (100 mg/kg/day) administered for 10 and 30 days in acute and chronic experiments, respectively. Conversely, the current study utilizes a lower dosage (1250 µg/day) due to the localized nature of the treatment. Notably, the study demonstrated superior efficacy for CUR-NE over non-nanotized CUR (CUR-S). In the acute experiment, CUR-NE could significantly extend the survival time of infected mice to 8.9 ± 0.87 days, compared to 5.6 ± 0.69 days in the NC group. Moreover, the mean counts of peritoneum tachyzoites in the CUR-NE group were significantly lower than in the NC group. In the chronic experiment, treatment with CUR-NE led to a decrease in the number and size of brain cysts in mice infected with *T. gondii* Tehran strain bradyzoites, compared to the NC group.

In our previous work, the exposure of *E. granulosus* to 1250 µg/ml of CUR-NE led to a 94% mortality rate after 60 min [31]. We showed that CUR-NE induced extensive disruptions in the tegumental surface of protoscolices and caused parasite death. In the present study, we also proved that CUR-NE is a potent anti-leishmanial agent against *L. major* promastigotes in both in vitro and in vivo. The flow cytometry results demonstrated that, in a dose-dependent manner, CUR-NE is a strong leishmanicidal agent against *L. major* promastigotes, killing 67.52% of the parasites at a concentration of 1250 µg/ml. Few studies have addressed the molecular bases for CUR anti-parasitic activity. It is assumed that this effect is probably related to the inhibition of and interference with cell signaling pathways and enzymes. It is possible that, similar to cancer cells, CUR causes cell cycle arrest and DNA damage in parasite cells [39]. Das et al. investigated the mechanism by which CUR is toxic to *L. donovani* promastigotes [15]. Parasites incubated with CUR were shown to be arrested at the G2/M phase of the cell cycle. By administering CUR, cytosolic calcium and ROS generation were enhanced, leading to mitochondrial membrane depolarization, cytosolic cytochrome c release, and multiple DNA damages. A recent study also proposed an inhibitory activity for CUR on histone acetyltransferase in *P. falciparum* [20].

Our study demonstrated the superiority of topical CUR-NE to topical CUR-S in reducing parasite burden and promoting lesion healing. Similarly, two other studies showed that intralesional injection of CUR-AgNPs and CUR-coated gold nanoparticles (CUR-AuNPs) administered at 20–60 mg/kg was able to effectively decrease the lesion size and parasite burden in a mouse model of CL [26, 34]. Notably, topical administration of CUR-NE offered a more convenient and less invasive approach than intralesional injection of CUR-AgNPs or CUR-AuNPs while maintaining acceptable effectiveness in reducing lesion size and parasite burden. It is possible that in the in vivo environment, along with anti-leishmanial effects, CUR manipulates macrophage nitric oxide (NO) and ROS generation and downregulates proinflammatory cytokines (e.g., tumor necrosis factor-alpha [TNF-α] and interleukin [IL]-1), thus attenuating tissue damage [40, 41]. Some studies attribute this anti-inflammatory activity of CUR to its ability to inhibit various intracellular molecules related to the nuclear factor kappa-light-chain-enhancer of activated B cells (NF-κB) signaling pathway. In addition, CUR facilitates macrophage polarization toward the M2 phenotype, which, unlike the M1 phenotype, mediates tissue repair, angiogenesis, and attenuates inflammation [42, 43].

An additional possible explanation is that secondary bacterial infections on CL lesions may prolong the healing process and cause further enlargement of the lesion. CUR is an effective and relatively broad-spectrum antibacterial agent [44]. It is active against the most common pathogens found in CL ulcers, including gram-positive bacteria, such as *Staphylococcus aureus* and *Enterococcus faecalis*, and gram-negative bacteria, such as *Escherichia coli* and *Pseudomonas aeruginosa* [45, 46]. Thus, CUR probably inhibits the superimposition of bacterial infections on CL lesions and leads to less tissue inflammation and faster wound healing. From a histological point of view, in addition to the modulatory effects of CUR on the inflammatory phase of wound healing [41], evidence shows that CUR facilitates fibroblast migration, collagen deposition, granulation tissue formation, and re-epithelialization during the proliferation phase [47]. Furthermore, in the remodeling phase, CUR enhances wound contraction, probably through upregulation of transforming growth factor-β (TGF-β) [48]. It is also noteworthy that in both in vitro and in vivo experiments, NE-no CUR had a minor anti-leishmanial effect against *L. major* promastigotes. This observation with NE particles may be due to some antioxidant and antimicrobial properties of soybean oil compounds, especially isoflavones [49].

Our study has a number of limitations. Although we showed a considerable therapeutic effect for CUR-NE on CL lesions, further studies are required for the assessment of drug safety. Moreover, we did not evaluate long-term adverse effects after exposure to CUR and CUR-NE. It is valuable to assess the long-term cellular effects of CUR, especially its effects on the cell cycle, as well as cosmetic aspects and superimposed bacterial complications. In addition, we employed the mean lesion size and parasite burden as indicators of wound healing; nonetheless, molecular studies are required to uncover the exact mechanism by which CUR resolves CL lesions and whether the compound can eradicate the parasite from local macrophages.

## Conclusion

The present study revealed the in vitro and in vivo therapeutic activity of CUR-NE. We showed an acceptable in vitro anti-leishmanial activity for CUR-NE against *L. major* promastigotes (MRHO/IR/75/ER), with the greatest effect at a concentration of 1250 µg/ml. In vivo experiments also indicated the promising properties of CUR-S and CUR-NE in healing and reducing parasite burden in CL lesions caused by *L. major* in mouse models. CUR-NE was more efficient than CUR-S, introducing a potential formula for therapeutic purposes. Future studies are required to identify molecular mechanisms as well as the pharmacologic and pharmacokinetic aspects of CUR-NE.

### Electronic supplementary material

Below is the link to the electronic supplementary material.

Supplementary Material 1

## Data Availability

All data generated or analyzed during this study is included in this published article. The raw data are available from the corresponding author upon reasonable request.
