# Supplementary material for: In vitro and in vivo anti-parasitic activity of curcumin nanoemulsion on Leishmania major (MRHO/IR/75/ER)
Source: BMC Complement Med Ther. 2024 Jun 18;24:238. doi: 10.1186/s12906-024-04522-1 (PMC11184741; doi:10.1186/s12906-024-04522-1)
Supplement: Supplementary file 1 — Supplementary Material 1 [file 12906_2024_4522_MOESM1_ESM.docx]

**Supplementary material**

**Table S1.** The mean, standard deviation, and median size of the lesions at different times in the six groups of mice.

| Kruskal-Wallis Test p-value | CUR-NE (injection) | CUR-S | NE-no CUR | CUR-NE (topical) | NC | PC |  | Group  Time |
| --- | --- | --- | --- | --- | --- | --- | --- | --- |
| 0.314 | 5.22 ± 1.67 | 4.45 ± 0.88 | 3.91 ± 0.38 | 4.73 ± 1.28 | 4.69 ± 0.26 | 4.37 ± 0.06 | Mean ± SD | Before treatment |
|  | 4.65 (2.42) | 4.40 (1.63) | 3.95 (0.71) | 4.80 (2.48) | 4.75 (0.45) | 4.40 (0.10) | Median (IQR) |  |
| 0.070 | 5.00 ± 1.85 | 4.15 ± 0.71 | 3.83 ± 0.47 | 4.16 ± 1.31 | 5.51 ± 0.76 | 3.62 ± 0.40 | Mean ± SD | After one week |
|  | 4.25 (2.97) | 3.95 (1.30) | 3.70 (0.80) | 4.05 (2.53) | 5.40 (1.48) | 3.85 (0.70) | Median (IQR) |  |
| 0.021* | 5.05 ± 2.14^a^ | 3.85 ± 0.52 | 3.81 ± 0.30 | 3.60 ± 1.41 | 6.29 ± 0.78 | 3.42 ± 0.28 | Mean ± SD | After two weeks |
|  | 4.00 (3.13) | 3.75 (1.00) | 3.60 (0.53) | 3.25 (2.63) | 6.10 (1.52) | 3.55 (0.45) | Median (IQR) |  |
| 0.005* | 4.85 ± 1.85 | 3.52 ± 0.49 | 3.82 ± 0.38 | 3.06 ± 1.34 | 7.23 ± 1.11 | 3.07 ± 0.16 | Mean ± SD | After three weeks |
|  | 4.05 (2.90) | 3.35 (0.88) | 3.45 (0.58) | 2.70 (2.35) | 6.60 (2.04) | 3.00 (0.05) | Median (IQR) |  |
| 0.003* | 4.80 ± 1.71 | 3.23 ± 0.59 | 3.85 ± 0.38 | 2.78 ± 1.28 | 8.56 ± 1.90 | 2.78 ± 0.23 | Mean ± SD | After four weeks |
|  | 4.20 (2.45) | 3.25 (1.10) | 3.50 (0.70) | 2.60 (2.45) | 8.10 (3.60) | 2.80 (0.25) | Median (IQR) |  |
|  | 0.066 | 0.001* | 0.18 | 0.001* | <0.001* | 0.022* |  | Friedman Test  p-value |

NC: negative control, PC: positive control, CUR-NE: curcumin nanoemulsion, NE-no CUR: nanoemulsion without curcumin, CUR-S: curcumin suspension, SD: standard deviation, IQR: interquartile range, *Statistically significant.
